# Immunoglobulin replacement therapy in patients with immunodeficiencies: impact of infusion method on patient-reported outcomes

**DOI:** 10.1186/s13223-022-00746-3

**Published:** 2022-12-24

**Authors:** Rajiv Mallick, Geneviève Solomon, Paul Bassett, Xiang Zhang, Palak Patel, Oleksandra Lepeshkina

**Affiliations:** 1 CSL Behring, King of Prussia, PA USA; 2 Association des Patients Immunodeficients du Québec, Québec, Canada; 3 Meridian HealthComms Ltd, Manchester, UK; 4 Formerly of CSL Behring, King of Prussia, PA USA; 5 Centre Hospitalier de l’Université Laval, Québec, Canada

**Keywords:** Immunodeficiency, Infusion method, Intravenous immunoglobulin (IVIg), Subcutaneous immunoglobulin (SCIg), Patient-reported outcomes, Treatment satisfaction questionnaire for medication 9 (TSQM-9), General health perception (GHP), Patient-reported outcomes measurement information system (PROMIS), Patient acceptable symptom state (PASS)

## Abstract

**Background:**

Understanding the impact of different immunoglobulin (Ig) infusion methods (intravenous [IVIg] and subcutaneous [SCIg]) upon treatment experience can potentially facilitate optimization of patient outcomes. Here, the perspective of patients with primary and secondary immunodeficiency diseases (PID and SID, respectively) receiving IVIg and SCIg was evaluated, in terms of treatment satisfaction, accounting for treatment history, using Association des Patients Immunodéficients du Québec (APIQ) survey data.

**Methods:**

The online APIQ survey (shared October 2020–March 2021) of patients with immunodeficiencies in Canada contained 101 questions on: Ig use, history, and detailed infusion characteristics; as well as structured patient-reported outcomes such as treatment satisfaction (via TSQM-9), symptom state (via PASS), general health perception (via GHP), and physical and mental function (via PROMIS). Adult respondents (≥ 18 years old) currently using Ig were compared by their current Ig infusion method (IVIg or SCIg cohort) overall, and in a sub-analysis, the IVIg cohort was compared with the SCIg cohort after stratification by respondents who started SCIg when naïve to Ig (‘SCIg naïve’) or with previous IVIg experience (‘SCIg switch’).

**Results:**

In total, 54 respondents currently used IVIg and 242 used SCIg. The average duration per infusion of a weekly SCIg infusion was significantly shorter compared with the average duration of a 3–4 weekly IVIg infusion (p < 0.001). The SCIg cohort was associated with significantly higher scores for the TSQM-9 *effectiveness* domain compared with the IVIg cohort. The scores for TSQM-9 *convenience* and *global satisfaction* domains were similar in the two cohorts. The SCIg cohort was also associated with a significantly higher proportion of respondents who were in an acceptable symptom state and a lower proportion who reported very poor or poor perception of health compared with the IVIg cohort. Further, the SCIg naïve subgroup was associated with significantly higher TSQM-9 *effectiveness* and *convenience* domain scores compared with the IVIg cohort, while there was no significant difference between the SCIg switch subgroup and the IVIg cohort in terms of *convenience*.

**Conclusions:**

A better understanding of how different IgRT administration methods impact treatment experience and satisfaction may assist with informed treatment decision making and ultimately further improvements in patient outcomes.

**Supplementary Information:**

The online version contains supplementary material available at 10.1186/s13223-022-00746-3.

## Background

Immunodeficiency diseases are disorders of the immune system that can predispose individuals to an increased rate and severity of infections, allergies, malignancies, and autoimmune diseases [1, 2]. Primary immunodeficiency diseases (PIDs) are a heterogeneous group of genetic disorders characterized by an intrinsic impairment in antibody production or function [1]. Secondary immunodeficiency diseases (SIDs) are caused by extrinsic factors that adversely affect the immune response including: malnutrition, treatment with glucocorticoids and immunomodulatory drugs, therapeutic intervention (e.g., hematopoietic stem cell transplantation [HSCT] and solid organ transplantation [SOT]), infectious diseases (e.g., human immunodeficiency virus [HIV]), environmental stress, extremes of age, and certain hematological malignancies [2, 3].

Despite their different pathogenesis, the clinical manifestations of PID and SID are usually similar, including recurrent or complicated infections of the upper and/or lower respiratory tract, caused by encapsulated bacteria [3, 4]. Treatments for PID and SID include prophylactic antibiotic therapy, immunosuppressants to improve symptom control in cases of autoimmune conditions, or immunoglobulin replacement therapy (IgRT) [2, 4].

Even with the availability of treatment, patients with immunodeficiencies can still experience a significant disease burden that can impact their physical function, emotional well-being, work productivity, disability, social interactions, and family life [5]. They have greater risk of lower school/work productivity, reduced life satisfaction, and experience anxiety or depressive symptoms in response to living with their chronic condition compared with healthy individuals [6, 7]. Regular long-term treatment regimens, while reducing disease burden, can introduce treatment-related burden in terms of interference with daily life, increased risk of adverse events (AEs), and acting as a constant reminder of the disease [8–11]. Studies have demonstrated that reduced treatment complexity and duration can decrease treatment burden and have a positive impact on patient compliance and overall quality of life [10].

Lifelong IgRT is the standard of care for patients with PID associated antibody deficiency and is known to reduce infections, morbidity, and mortality [12, 13]. There is also growing evidence to support the use of IgRT in patients with SID [14–17]. IgRT can be administered either intravenously (IVIg) or subcutaneously (SCIg), with both routes of administration reported as effective and well-tolerated [8, 18]. IVIg was the most common type of infusion method of IgRT during the 1980s and 1990s [18]. However, over time, SCIg has increasingly established itself as a well-tolerated and effective treatment that is preferred by many patients due to the reduced incidence of systemic AEs, more stable serum immunoglobulin G (IgG) levels, flexibility in scheduling, and its comparative ease of administration, at home or in a clinic compared with IVIg [18–20]. Patients who self-administer SCIg at home have reported improved quality of life through increased flexibility, freedom, feeling of self-responsibility, and less absence from work or school compared with IVIg [21–23]. During the global coronavirus disease 2019 (COVID-19) pandemic, patients who were identified as being extremely clinically vulnerable were encouraged to receive treatment at home, where possible, in many countries [24, 25]. Home-administration of SCIg provided patients with immunodeficiencies the opportunity to access IgRT without visiting a healthcare setting, thereby mitigating the risk of nosocomial exposure to COVID-19 [24].

Multiple infusion method options for IgRT allow treatment regimens to be tailored to suit an individual patient’s needs and circumstances [8]. Indeed, the choice of modality of administration can have major implications for patient well-being. A survey of patients with PID on their quality of life and views on IgRT found that many patients have a desire for less frequent infusions, shorter infusion duration, the ability to self-administer at home, and fewer needle sticks compared with their current treatment regimen, highlighting the importance of providing access to different infusion method options [19].

After transitioning from IVIg to SCIg, several studies have demonstrated that patients perceived significant improvements in health status and immunoglobulin (Ig)-specific perceptions of health related quality of life (HRQoL), as measured by the Short Form 36 Health Status Questionnaire (SF-36) and Life Quality Index (LQI) questionnaire [22, 26, 27] respectively. One recent study of patients switching infusion methods demonstrated improvements specifically in several LQI items—patient convenience, comfort, independence, treatment schedule flexibility, pleasantness of treatment setting, and less disruption of daily activities, when patients transitioned from IVIg to SCIg [28]. Furthermore, these improvements were maintained over a follow-up period of 3 years [28].

Switching from IVIg to home-based self-administered SCIg has also led to improved perceptions of general health in both adult and pediatric patients with PID (evaluated over 12 months), including marked improvements in bodily pain and vitality assessments [29]. In addition, the number of days of work or school missed by patients over the 12 month follow up period were greatly reduced, and parents of children with PID had improved perceptions of personal and family activities [29]. Nevertheless, favorable patient perceptions of SCIg infusion satisfaction have been partly also based on association with better training experience, infusion efficiency, age, and longer treatment experience, highlighting the importance of accompanying patient education [30]. In general, patient preference assessments have highlighted the importance of continually offering patients information and a choice of IgRT infusion options; in addition to training, clinical support, and shared decision making, even if patients have been satisfied on one type of IgRT infusion method for many years [30–32].

Despite the improvements in quality of life associated with switching from IVIg to SCIg, some patients may be discouraged because of perceived inconvenience, concerns about AEs at home, and a fear of needles [33]. A better understanding of the impact of different IgRT infusion methods on treatment satisfaction and well-being will allow for more evidence-based decision making, help guide best practice for IgRT, and facilitate optimization of patient outcomes. In addition, due to variations in the availability of and access to IgRT infusion methods, particularly during the COVID-19 era, there is also a need for region-specific analysis of patient perceptions associated with IgRT infusion methods.

Here, perspectives of patients with PID and SID receiving IVIg and SCIg are evaluated, accounting for IgRT treatment history and patient demographics. Based upon these findings, we provide evidence-based recommendations for improving IgRT infusion-related experiences for patients with immunodeficiencies.

## Methods

### Data source

Using the Canadian Immunodeficiencies Patient Organization (CIPO)—APIQ database, patients with immunodeficiencies in Canada were contacted via email regarding an incentivized online survey between October 2020 and March 2021. The survey contained 101 questions on IgRT use and respondent perceptions (Additional file 1), including: demographic characteristics, reasons for choosing an IgRT infusion method, infusion characteristics, IgRT history, details of switching between IVIg and SCIg, SCIg training experiences, and structured patient-reported outcomes (PROs). PROs included (i) the Treatment Satisfaction for Medication Questionnaire (TSQM-9) [34], (ii) Patient Acceptable Symptom State (PASS) [35], to measure symptom status, (iii) General Health Perception (GHP) [36], to measure overall HRQoL, and (iv) the Patient-Reported Outcomes Measurement Information System (PROMIS) [37] two-item Global Physical Health (GPH-2) and two-item Global Mental Health (GMH-2) scales, respectively [38].

### Study exclusion criteria and study cohorts

**Fig. 1:**
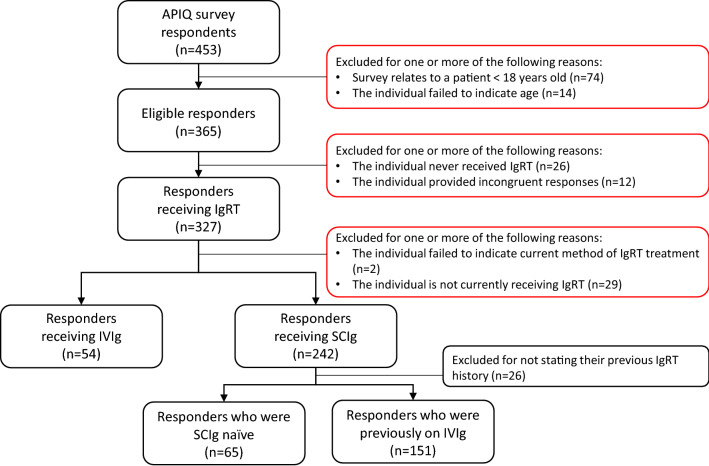
Criteria used to include respondents in the study. *APIQ* Associatin des Patients Immunodéficients du Québec, *IgRT* immunoglobulin replacement therapy, *IVIg* intravenous immunoglobulin G, *SCIg* subcutaneous immunoglobulin

Respondents were excluded using the following criteria: < 18 years old or failure to indicate age, were not currently receiving IgRT, incomplete or incongruent responses (i.e., those with incompatible responses such as selecting currently receiving SCIg but citing an IVIg product). Respondents were stratified by their current IgRT infusion method into an IVIg or SCIg cohort (Fig. 1). The SCIg cohort was further stratified by their IgRT history: respondents who were naïve to Ig (‘SCIg naïve’) or respondents who had switched from IVIg to SCIg (‘SCIg switch’) (Fig. 1). These subgroups were compared separately with the IVIg cohort.

### Outcomes

*Treatment satisfaction* was the primary concept of interest that would be amenable to differences in modes of IgRT administration. This outcome was assessed using a modified version of the TSQM-9, which measured patients’ satisfaction with medication. In this survey, the instructions to the TSQM-9 asked respondents to focus on the infusion process in their responses, but the wording of the items (questions) themselves was not modified. The TSQM-9 was scored on a verbal rating scale anchored from one to five or seven depending on the question (1–5, where 1 = extremely poor experience/perception and 5 = extremely satisfied experience/perception; 1–7, where 1 = extremely poor experience/perception and 7 = extremely satisfied experience/perception). Raw scores were transformed for each TSQM-9 domain to a 0–100 scale from worst to best.

Patient symptom and overall well-being were secondary concepts of interest. Patient *symptom status* was measured using PASS, a single-item, dichotomous measure of patient acceptable symptom state based on a single question, “Considering all the different ways your disease is affecting you, if you would stay in this state for the next months, do you consider that your current state is satisfactory?” [35]. Patients could respond in the affirmative (yes) or in the negative (no).

Overall well-being was encapsulated in the concept of *perceived health status*, measured in terms of the single-item GHP question, “Would you describe your current health status as excellent, very good, good, fair, poor, or very poor?” and a 6-point Likert scale scoring system (1 = excellent, 2 = very good, 3 = good, 4 = fair, 5 = poor, 6 = very poor) [36]. We dichotomized responses by combining the excellent, very good, good, and fair categories vs. poor, or very poor for ease of interpretation.

Patient physical function and mental health were assessed using the PROMIS GPH-2 and GMH-2 for adult responses, respectively [38]. Summed scores on each were transformed to corresponding PROMIS T-scores using previously published concordance tables [39].

### Statistical analyses

Transformed TSQM-9 scores, GHP scores, PASS percentages, and PROMIS GPH-2 and GMH-2 T-scores were initially compared overall between the IgRT infusion cohorts (IVIg vs. SCIg), and subsequently stratified by SCIg history (SCIg naïve vs. IVIg; SCIg switch vs. IVIg) subgroups. Categorical variables were compared between infusion cohorts using the chi-squared test, and continuous variables were compared between groups using the unpaired t-test if found to be normally distributed, or the Mann-Whitney test otherwise. Analysis of variance (ANOVA) post-hoc tests were used when comparing three groups.

## Results

In total, 72.2% (327/453) of respondents to the APIQ survey indicated ever having received IgRT and were included in our analysis (Fig. 1). Of these, 325 (99.4%) were residents of Québec. Of the 327 respondents, 54 (16.5%) indicated IVIg as their current infusion method and 242 (74.0%) indicated SCIg as their current infusion method. The remaining 31 (9.5%) respondents were excluded due to either a failure to indicate their current IgRT infusion method (0.6% [n = 2]), or they indicated that they were not currently receiving IgRT (8.7% [n = 29]). A subsequent separate analysis focuses on the comparison of SCIg drug packaging options—vials vs. pre-filled syringes.

### Respondent characteristics

Respondent characteristics of the IVIg and SCIg cohorts are summarized in Table 1. The median (interquartile range [IQR]) age of all respondents was 59 (37, 67) years. There were significantly more females in the SCIg cohort and more males in the IVIg cohort (p = 0.01; Table 1). Respondents in the IVIg cohort were significantly more likely to have received antibiotic treatment since starting their IgRT compared with those in the SCIg cohort (78.6% [n = 33] vs. 60.4% [n = 139] respectively, p = 0.03; Table 1). Respondents in the IVIg cohort reported a greater unfavorable impact of their treatment regimen on work/school attendance compared with respondents in the SCIg cohort (33.3% [n = 18] vs. 8.3% [n = 20] respectively, p < 0.001; Table 1).

**Table 1:** Patient characteristics, by IgRT modality and history

Respondent characteristics		IVIg cohort (n = 54) (A)		SCIg cohort (n = 242) (B)		SCIg naïve subgroup (n = 65) (C)		SCIg switch subgroup (n = 151) (D)		p values		
		Summary	n	Summary	n	Summary	n	Summary	n	A vs. B	A vs. C	A vs. D
Age (years), median [IQR]		60 [37, 67]	54	59 [48, 67]	242	64 [52, 68]	65	57 [45, 64]	151	0.43	0.13	0.94
Age at diagnosis (years), median [IQR]		38 [18, 60]	54	45 [27, 60]	239	54 [40, 64]	63	40 [23, 54]	150	0.23	**0.003**	0.79
Gender, n (%)												
Female		24 (44.4%)	54	151 (63.0%)	240	35 (54.7%)	64	94 (62.7%)	150	**0.01**	0.27	**0.02**
Male		30 (55.6%)		89 (37.1%)		29 (45.3%)		56 (37.3%)				
Weight (kg)		74.2 ± 15.1	47	75.8 ± 17.4	216	80.5 ± 15.3	53	73.8 ± 16.3	138	0.56	0.05	0.89
Underlying condition, n (%)												
CVID		21 (51.2%)	41	85 (45.0%)	189	20 (41.7%)	48	56 (45.5%)	123	0.35	0.32	0.23
IgG Sub		9 (22.0%)		48 (25.4%)		10 (20.8%)		35 (28.5%)				
DGS		2 (4.9%)		8 (4.2%)		4 (8.3%)		3 (2.4)				
SID		1 (2.4%)		22 (11.6%)		7 (14.6%)		14 (11.4%)				
Other*		8 (19.5%)		26 (13.8%)		7 (14.6%)		15 (12.2%)				
Years since diagnosis, n (%)												
< 2 years		3 (5.6%)	54	16 (6.7%)	239	10 (15.9%)	63	5 (3.3%)	150	0.24	** < 0.001**	0.74
2–9 years		20 (37.0%)		116 (48.5%)		41 (65.1%)		60 (40.0%)				
≥ 10 years		31 (57.4%)		107 (44.8%)		12 (19.1%)		85 (56.7%)				
Time on IgG, n (%)												
< 1 year		1 (1.9%)	54	11 (4.6%)	242	7 (10.8%)	65	3 (2.0%)	151	0.6	** < 0.001**	0.82
1–2 years		5 (9.3%)		35 (14.5%)		10 (15.4%)		21 (13.9%)				
2–3 years		7 (13.0%)		31 (12.8%)		13 (20.0%)		13 (8.6%)				
4–6 years		11 (20.4%)		55 (22.7%)		24 (36.9%)		24 (15.9%)				
≥ 6 years		30 (55.6%)		110 (45.5%)		11 (16.9%)		90 (59.6%)				
Current treatment experience, n (%)												
< 2 years		4 (8.5%)	47	35 (14.7%)	238	12 (18.5%)	65	20 (13.5%)	148	**0.02**	** < 0.001**	0.23
2–9 years		23 (48.9%)		148 (62.2%)		46 (70.8%)		84 (56.8%)				
≥ 10 years		20 (42.6%)		55 (23.1%)		7 (10.8%)		44 (29.7%)				
Antibiotics before IgG, n (%)												
No		29 (70.7%)	41	157 (73.7%)	213	48 (81.4%)	59	94 (71.2%)	132	0.69	0.21	0.95
Yes		12 (29.3%)		56 (26.3%)		11 (18.6%)		38 (28.8%)				
Antibiotics since starting IgG, n (%)												
No		9 (21.4%)	42	91 (39.6%)	230	29 (46.0%)	63	53 (37.1%)	143	**0.03**	**0.01**	0.06
Yes		33 (78.6%)		139 (60.4%)		34 (54.0%)		90 (62.9%)				
Missed work/school due to treatment regimen, n (%)												
No		36 (66.7%)	54	221 (91.7%)	2	63 (96.7%)	65	134 (89.3%)	150	** < 0.001**	** < 0.001**	** < 0.001**
Yes		18 (33.3%)		20 (8.3%)		2 (3.1%)		16 (10.7%)				

Data were compared using Mann–Whitney test, unpaired t-test or chi-square test. Significant p-values are highlighted in bold. *Other indications are: XLA (n = 9), SCID (n = 8), CGD (n = 1), specific antibody deficiency (n = 4), idiopathic autoimmune hemolytic anemia (n = 1), autoimmune disease (n = 2), hypogammaglobulinemia (n = 5), CLL (n = 1), myasthenia gravis (n = 1), and Waldenstrom macroglobulinemia (n = 2). *CGD* chronic granulomatous disease, *CLL* chronic lymphocytic leukemia, *CVID* common variable immune deficiency, *DGS* DiGeorge syndrome, *GHP* general health perception, *GMH-2* global mental health 2, *GPH-2* global physical health 2, *IgG* immunoglobulin G, *IgG Sub* immunoglobulin subclass deficiency, *IgRT* immunoglobulin replacement therapy, *IQR* interquartile range, *IVIg* intravenous immunoglobulin, *kg* kilogram, *SCID* severe combined immunodeficiency, *SCIg* subcutaneous immunoglobulin, *SD* standard deviation, *SID* secondary immunodeficiencies, *XLA* X-linked agammaglobulinemia

### Comparison of IVIg and SCIg users

#### Choice of initial IgRT infusion method and modality

The distribution of who is responsible for deciding patients’ infusion method varied significantly between the two infusion method cohorts. The proportion of respondents who stated the prescriber as being responsible was highest in both the IVIg and SCIg cohorts, but more so in the SCIg cohort (52.1% [n = 25] vs. 67.8% [n = 164], respectively), followed by the medical facility (25.0% [n = 12] vs. 22.3% [n = 54], respectively). The proportion of patients themselves being responsible for choosing the IgRT infusion method was higher in the IVIg cohort compared with the SCIg cohort (22.9% [n = 11] vs. 9.9% [n = 24], respectively).

**Fig. 2:**
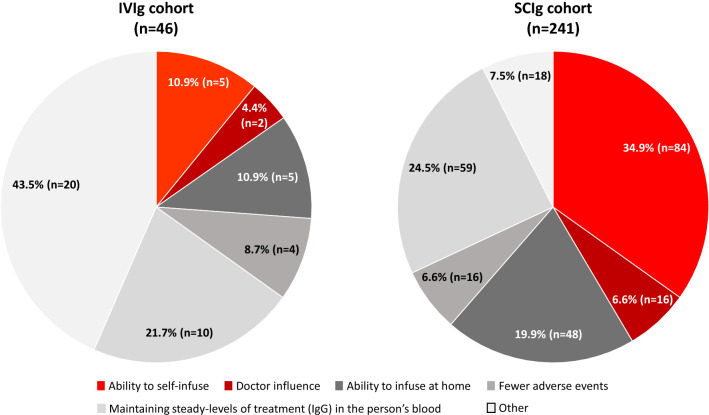
Respondents’ reasons for choosing an IgRT infusion method, by infusion method (IVIg and SCIg). *IgG* immunoglobulin G, *IgRT *immunoglobulin replacement therapy, *IVIg* intravenous immunoglobulin, *SCIg* subcutaneous immunoglobulin

To understand the factors taken into consideration by patients when choosing an infusion method, information on the three most important reasons a method was chosen was collected for the two cohorts (Fig. 2). Overall, respondents in the IVIg and SCIg cohorts varied significantly with respect to what they considered the most important factors (p < 0.001). The ability to self-infuse was the most common factor for respondents in the SCIg cohort (34.9% [n = 84]), whilst the most common factor in the IVIg cohort was steady levels of serum (blood) IgG (21.7% [n = 10]) (Fig. 2).

Only relatively small numbers of respondents from both the IVIg and SCIg cohorts changed their infusion method preference on account of the COVID-19 pandemic, with no significant difference between the two cohorts (10.8% [n = 4] vs. 8.9% [n = 20], respectively, p = 0.71). This survey found that only one IVIg respondent and 10 SCIg respondents changed their current infusion method on account of the pandemic, with no significant difference in the proportions between the two cohorts (p = 0.53). Respondents in the SCIg cohort were more likely to state the risk of infection as being the reason for having changed their infusion method compared with the IVIg cohort (60.0% [n = 12] vs. 0.0% [n = 0], respectively, p = 0.03). For those who did not change infusion method, the majority of respondents from both the IVIg and SCIg cohorts perceived no impact on their treatment of remaining with their current infusion method during the pandemic, with no significant difference between the two cohorts (90.7% [n = 39] vs. 94.6% [n = 211], respectively, p = 0.32).

#### Infusion characteristics

To assess the burden of treatment infusion, information on infusion duration, frequency, and location was collected for the two infusion method cohorts (Table 2). The median duration of the actual infusion time was significantly shorter in the SCIg cohort compared with the IVIg cohort (60 vs. 165 min, respectively, p < 0.001; Table 2). However, the average frequency of infusions was significantly higher for the SCIg cohort compared with the IVIg cohort (p < 0.001, Table 2), with the majority of the SCIg cohort (74.4% [n = 163]) receiving weekly infusions, whilst the majority of the IVIg cohort (75.9% [n = 22]) received infusions every 4 weeks (Table 2). On average, the SCIg cohort also had significantly quicker infusion preparation time and post-infusion clean up time compared with the IVIg cohort (15 vs. 30 min, respectively, p < 0.001, and 5 vs. 15 min respectively, p = 0.005, respectively; Table 2).

**Table 2:** IgRT infusion characteristics: IVIg vs. SCIg

Infusion characteristics	IVIg cohort (n = 54)		SCIg cohort (n = 242)		p-value
	Summary	n	Summary	n	
Frequency of treatment, n (%)					
Every day	0 (0.0%)	29	2 (0.9%)	219	** < 0.001**
Two per week	0 (0.0%)		15 (6.9%)		
Three per week	0 (0.0%)		10 (4.6%)		
Weekly	1 (3.5%)		163 (74.4%)		
Every 2 weeks	1 (3.5%)		29 (13.2%)		
Every 3 weeks	5 (17.2%)		0 (0.0%)		
Every 4 weeks	22 (75.9%)		0 (0.0%)		
Travel time (mins)* (median [IQR])	40 [30, 90]	27	60 [30, 75]	195	N/A
Trips to hospital/pharmacy to pick up SCIg supplies per month, n (%)					
< 1	N/A	N/A	119 (58.1%)	205	N/A
1			80 (39.0%)		
2			2 (1.0%)		
3			1 (0.5%)		
4			2 (1.0%)		
> 4			1 (0.5%)		
Location of infusions, n (%)					
Hospital	27 (96.4%)	28	N/A	N/A	N/A
Home	1 (3.6%)				
Infusion preparation time (mins) (median [IQR])	30 [15, 30]	27	15 [10, 20]	207	** < 0.001**
Actual infusion time (mins) (median [IQR])	165 [126, 255]	28	60 [40, 90]	207	** < 0.001**
Post-infusion clean up time (mins) (median [IQR])	15 [6, 20]	27	5 [5, 10]	201	**0.005**

Data were compared using a Mann-Whitney test. ^*^Travel time to infusion center for IVIg respondents, travel time to the pharmacy/hospital for SCIg respondents. Significant p-values are highlighted in bold. *IgRT* immunoglobulin replacement therapy, *IQR *interquartile range, *IVIg* intravenous immunoglobulin, *mins* minutes, *N/A* not applicable, *SCIg* subcutaneous immunoglobulin

The median (IQR) travel time for the IVIg cohort to travel back and forth to their infusion center was 40 (30, 90) minutes per trip, of which most had to do every 4 weeks (75.9% [n = 22]). The majority (58.5% [n = 119]) of SCIg respondents made fewer than one trip per month to the pharmacy or hospital to collect SCIg supplies (Table 2). When a trip was made, the median (IQR) travel time for the SCIg cohort to travel back and forth to the pharmacy or hospital to collect SCIg supplies was 60 (30, 75) minutes per trip (Table 2).

#### Patient-reported treatment satisfaction (TSQM-9)

##### TSQM-9 *effectiveness*

**Fig. 3:**
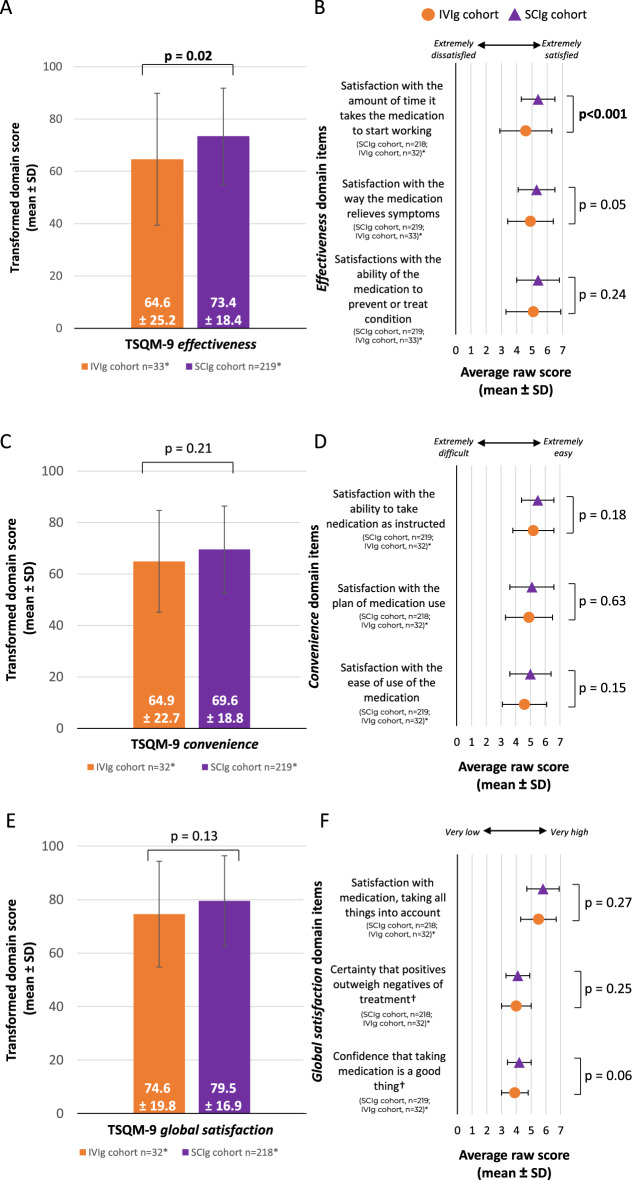
Perceived treatment satisfaction in the SCIg and IVIg cohorts. **A** Transformed TSQM-9 *effectiveness* domain scores and **B** raw scores from the corresponding TSQM-9 domain items. **C** Transformed TSQM-9 *convenience* domain scores and **D** raw scores from the corresponding TSQM-9 domain items. **E** Transformed TSQM-9 *global satisfaction* domain scores and **F** raw scores from the corresponding TSQM-9 domain items. *n numbers vary due to missing respondent data for various survey questions. Transformed domain scores are on a 0–100 scale from worst to best and the raw scores are on a one to five or seven scale from extremely dissatisfied to extremely satisfied. Continuous variables were compared using an unpaired t-test and the significant p values are in bold. †Domain items rated on a scale from 1–5. *IVIg* intravenous immunoglobulin, *SCIg* subcutaneous immunoglobulin, *SD* standard deviation, *TSQM-9* treatment satisfaction questionnaire for medication 9

Respondents in the SCIg cohort scored significantly higher in the TSQM-9 *effectiveness* domain compared with those in the IVIg cohort (p = 0.02, Fig. 3A). When examining item specific evidence on the TSQM-9* effectiveness* domain, the SCIg cohort was associated with significantly greater satisfaction with *the amount of time it takes the medication to start working* compared with the IVIg cohort (p < 0.001, Fig. 3B).

##### TSQM-9 *convenience*

There was no significant difference between the SCIg and IVIg cohorts in the score for the TSQM-9 *convenience* domain (p = 0.21, Fig. 3C). Similarly, there was no significant difference between the two cohorts in the scores for any item specific evidence on the TSQM-9 *convenience* domain (all p > 0.05, Fig. 3D).

##### TSQM-9 *global satisfaction*

There was no significant difference between the SCIg and IVIg cohorts in the score for the TSQM-9 *global satisfaction* domain (p = 0.13, Fig. 3E). Similarly, there was no significant difference between the two cohorts in the scores for any item specific evidence on the TSQM-9 *global satisfaction* domain (all p > 0.05, Fig. 3F).

#### Other patient-reported outcomes

**Fig. 4:**
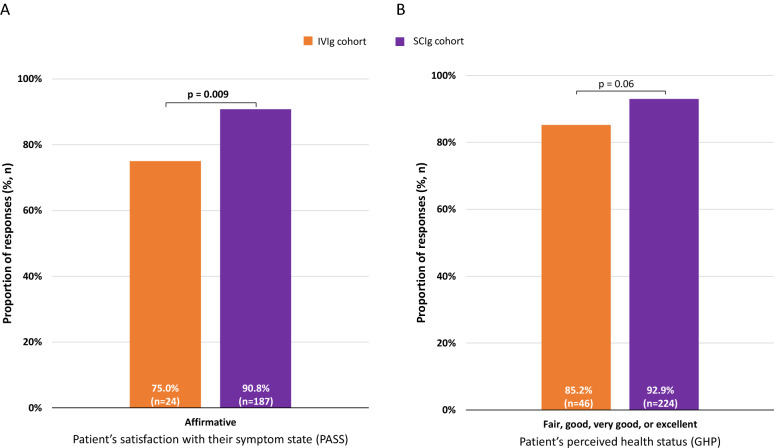
Respondent symptom state and perceived health status in the SCIg and IVIg cohorts. **A** Proportion of respondents who responded ‘affirmative’ to whether they were at an acceptable symptom state (measured using PASS) and **B** proportion of respondents who described their current health status as ‘fair’, ‘good’, ‘very good’ or ‘excellent’ (measured using GHP). The complementary response category for PASS was ‘negative’ and for GHP were ‘poor’ or ‘very poor’. Significant p-values are highlighted in bold. *GHP* general health perception, *IVIg* intravenous immunoglobulin, *PASS* patient acceptable symptom state, *SCIg* subcutaneous immunoglobulin

The SCIg cohort had a significantly higher proportion of patients in the PASS-affirmative category (i.e., responded they were at an acceptable symptom state) compared with the IVIg cohort (90.8% [n = 187] vs. 75.0% [n = 24], p = 0.009; Fig. 4A). In addition, measurement of overall well-being in terms of GHP revealed that a lower proportion of SCIg respondents reported that they were in very poor or poor health compared with the IVIg cohort (7.1% [n = 17] vs. 14.8% [n = 8], p = 0.06; Fig. 4B). There were no significant differences between the IVIg and SCIg cohorts for the mean (± standard deviation [SD]) PROMIS GPH-2 T-scores (46.3 ± 8.6 [n = 31] vs. 47.0 ± 7.5 [n = 218], respectively) and GMH-2 T-scores (51.3 ± 10.5 [n = 31] vs. 51.1 ± 7.9 [n = 218], respectively)**.**

### Sub analysis of SCIg patients based upon their IgRT history

Of the SCIg users (n = 242), 65 had never previously received IgRT and were assigned to the ‘SCIg naïve’ subgroup. Another 151 SCIg users had previously received IVIg and were assigned the ‘SCIg switch’ subgroup. Respondent characteristics of the SCIg naïve and SCIg switch subgroup cohorts, in relation to the IVIg cohort, are shown in Table 1. Time since immunodeficiency diagnosis was significantly lower in the SCIg naïve cohort compared with the IVIg cohort (p < 0.001, Table 1), with only 19.1% (n = 12) in the SCIg naïve cohort having at least 10 years since diagnosis compared with 57.4% (n = 31) in the IVIg cohort (Table 1). Similarly, the amount of experience on their current treatment was also significantly lower for the SCIg naïve cohort, with only 10.8% (n = 7) of this cohort having over 10 years’ experience with IgRT, compared with 42.6% (n = 20) of the IVIg cohort (p < 0.001, Table 1). Both the SCIg naïve and SCIg switch cohorts were less likely to miss days at work/school compared with the IVIg cohort (p < 0.001 for both comparisons, Table 1). All other comparisons of characteristics in the IVIg cohort and the two subgroups were not significant.

#### Switching IgRT infusion method

Of the patients who switched from IVIg to SCIg (SCIg switch group), the most common stakeholder responsible for choosing whether the respondent switched was the prescriber (70.0% [n = 101]). The most common reasons stated by respondents for switching from IVIg to SCIg were recommendations made by the prescriber, followed by greater scheduling convenience (stated by 52.1% [n = 73] and 39.3% [n = 55] of respondents, respectively). Other key reasons for the switch stated by respondents included: (i) maintaining steady levels of treatment (IgG) in the person’s blood (37.1% [n = 52]), (ii) avoiding IVIg associated AEs (29.3%, [n = 41]), (iii) avoiding travel for IVIg infusions (20.0%, [n = 28]), and (iv) difficulties with venous access (17.1% [n = 24]).

There was generally a positive effect of switching from IVIg to SCIg on quality of life and physical and mental health. Of the respondents who switched from IVIg to SCIg, 81.5% (n = 101) reported an improved or substantially improved quality of life and 33.1% (n = 41) reported a substantial improvement. Improved or substantially improved physical health was reported by 58.1% (n = 72) of respondents after switching from IVIg to SCIg and 44.4% (n = 55) reported improved or substantially improved mental health, with only 0.8% (n = 1) and 4.0% (n = 5) reporting worse physical and mental health respectively. Treatment satisfaction, treatment compliance, and productivity were also reported to be improved after switching from IVIg to SCIg. Two-thirds (66.7% [n = 82]) of respondents reported improved or substantially improved treatment satisfaction, 29.3% (n = 36) of whom reported a substantial improvement. Very few patients (3.3% [n = 4]) reported a worse compliance to treatment after their switch, and 37.4% (n = 46) reported improved or substantially improved compliance. Almost three-quarters (72.6% [n = 90]) of respondents reported improved or substantially improved productivity, 27.4% (n = 34) of whom reported a substantial improvement.

Only a small number of respondents (n = 9) switched from SCIg to IVIg. In all instances, the patient initiated the discussion to switch to IVIg, most commonly due to difficulty of administration (66.7% [n = 6]). Other reasons for the switch stated by respondents included AEs (44.4% [n = 4]), high dosing frequency (44.4% [n = 4]), long infusion times (44.4% [n = 4]), treatment failing to work as desired (33.3% [n = 3]), treatment complexity (11.1% [n = 1]), and treatment being a reminder of the disease (11.1% [n = 1]).

#### SCIg training experience

To understand the factors associated with training experience and satisfaction, information regarding training characteristics of the two SCIg subgroups were collected (Table 3). The statistical comparisons revealed no significant differences between the two subgroups for any of the measures examined (p > 0.05, Table 3). For both the SCIg naïve and SCIg switch cohorts, three-quarters of respondents found training easy or very easy (76.2% [n = 48] and 75.4% [n = 101], respectively; Table 3). On average, half of respondents had only one training session (52.4% [n = 33] in the SCIg naïve subgroup, and 50.8% [n = 68] in the SCIg switch subgroup; Table 3), with a further 25.4% (n = 16) in the SCIg naïve and 28.4% (n = 35) of the SCIg switch cohorts having two sessions (Table 3). The median (IQR) length of SCIg training was 2 (1, 2) hours in the SCIg naïve subgroup and 1.5 (1, 2) hours in the SCIg switch subgroup (Table 3). The most common training location was in hospitals for both the SCIg naïve and SCIg switch subgroups (61.9% [n = 39] and 59.7% [n = 80], respectively; Table 3); and a nurse was the most common provider of training for both the SCIg naïve and SCIg switch subgroups (62.9% [n = 39] and 57.1% [n = 76], respectively; Table 3). In both subgroups, the training was typically conducted by a hospital nurse (62.9% and 57.1% in the SCIg naïve and SCIg switch subgroups, respectively), followed by a pharmaceutical manufacturer’s training program nurse (30.7% and 30.8% in the SCIg naïve and SCIg switch subgroups, respectively; Table 3). Overall, the trainer was graded as knowledgeable or very knowledgeable by over 95% of respondents (Table 3), and a similar proportion of respondents ranked the trainer as competent or very competent (Table 3). The majority of respondents (SCIg naïve, 88.9% [n = 56] and SCIg switch, 86.5% [n = 115]) felt they were confident or very confident in self-administering SCIg following training (Table 3). Overall, over 95% of respondents in both subgroups were satisfied with their training, the majority of which were very satisfied (Table 3). Less than 1% of patients in both subgroups reported any barriers to training (Table 3). When asked what their greatest training concern was, the most commonly reported answer was inserting the needle (SCIg naïve, 42.9% [n = 27] and SCIg switch, 43.9% [n = 58], Table 3).

**Table 3:** Characteristics of respondent training for SCIg self-infusion by IgRT history

SCIg training characteristics	SCIg naïve subgroup (n = 65)		SCIg switch subgroup (n = 151)		p-value
	Summary	n	Summary	n	
Ease of SCIg training, n (%)					
Very difficult	1 (1.6%)	63	4 (3.0%)	134	0.88
Difficult	6 (9.5%)		8 (6.0%)		
Neither	8 (12.7%)		21 (15.7%)		
Easy	24 (38.1%)		52 (38.8%)		
Very easy	24 (38.1%)		49 (36.6%)		
Number of SCIg training sessions, n (%)					
1	33 (52.4%)	63	68 (50.8%)	134	0.9
2	16 (25.4%)		38 (28.4%)		
3	9 (14.3%)		17 (12.7%)		
4	3 (4.8%)		4 (3.0%)		
5	1 (1.6%)		1 (0.8%)		
> 5	1 (1.6%)		6 (4.5%)		
SCIg training locations, n (%)					
Doctors’ office	0 (0.0%)	63	5 (3.7%)	134	0.36
Home	19 (30.2%)		44 (32.8%)		
Hospital	39 (61.9%)		80 (59.7%)		
Infusion center	2 (3.2%)		3 (2.2%)		
Other^*^	3 (4.8%)		2 (1.5%)		
Type of SCIg trainer, n (%)					
Doctor’s office staff	4 (6.5%)	62	13 (9.8%)	133	0.7
Hospital nurse	39 (62.9%)		76 (57.1%)		
Hizentra CARE nurse	19 (30.7%)		41 (30.8%)		
Other^†^	0 (0.0%)		3 (2.3%)		
Trainer knowledge, n (%)					
Very naïve	0 (0.0%)	63	0 (0.0%)	132	0.33
Naive	2 (3.2%)		2 (1.5%)		
Neither	0 (0.0%		1 (0.8%)		
Knowledgeable	3 (4.8%)		14 (10.6%)		
Very knowledgeable	58 (92.1%)		115 (87.1%)		
Trainer competency, n (%)					
Very incompetent	0 (0.0%)	62	1 (0.8%)	133	0.09
Incompetent	1 (1.6%)		2 (1.5%)		
Neither	0 (0.0%)		1 (0.8%)		
Competent	2 (0.0%)		13 (9.8%)		
Very competent	59 (95.2%)		116 (87.2%)		
Self-administration confidence, n (%)					
Very uncertain	0 (0.0%)	63	1 (0.8%)	133	0.41
Uncertain	5 (7.9%)		6 (4.5%)		
Neither	2 (3.2%)		11 (8.3%)		
Confident	20 (31.8%)		48 (36.1%)		
Very confident	36 (57.1%)		67 (50.4%)		
Training					
satisfaction, n (%)					
Very dissatisfied	0 (0.0%)	63	0 (0.0%)	133	0.06
Dissatisfied	0 (0.0%)		3 (2.3%)		
Neither	1 (1.6%)		3 (2.3%)		
Satisfied	7 (11.1%)		26 (19.6%)		
Very satisfied	55 (87.3%)		101 (75.9%)		
Perceived barriers to training, n (%)					
No	57 (100%)	57	120 (99.2%)	121	1
Yes	0 (0.0%)		1 (0.8%)		
Length of training (hours) (median [IQR])	2 [1, 2]	60	1.5 [1, 2]	127	0.52
Greatest training concern^‡^, n (%)					
Drawing the drug	6 (9.5%)	63	19 (14.4%)	132	0.82
Inserting the needle	27 (42.9%)		58 (43.9%)		
Operating the pump	2 (3.2%)		2 (1.5%)		
Priming the tube	3 (4.8%)		7 (5.3%)		
Other	1 (1.6%)		1 (0.8%)		
No concerns	24 (38.1%)		45 (34.1%)		

Data were compared using a Mann-Whitney test (ease of SCIg training, number of SCIg training sessions) or Fisher’s exact test (SCIg training location, type of SCIg trainer). Significant p values are in bold. ^*^Other training locations: Institut de rescherches Clinique de Montreal (n = 4) and a local community services centre (n = 1). ^†^Other trainers: home health/specialty pharmacy staff (n = 1), one PATH nurse (n = 1), and ProCare (n = 1). ^‡^Other concerns: tip-to-tip transfer (n = 2) and applying needles to tubing (n = 2). *IgRT* immunoglobulin replacement therapy, *IQR *interquartile range, *SCIg *subcutaneous immunoglobulin

#### Patient-reported treatment satisfaction (TSQM-9): differences between the two SCIg subgroups and the IVIg cohort

##### TSQM-9 *effectiveness*

**Fig. 5:**
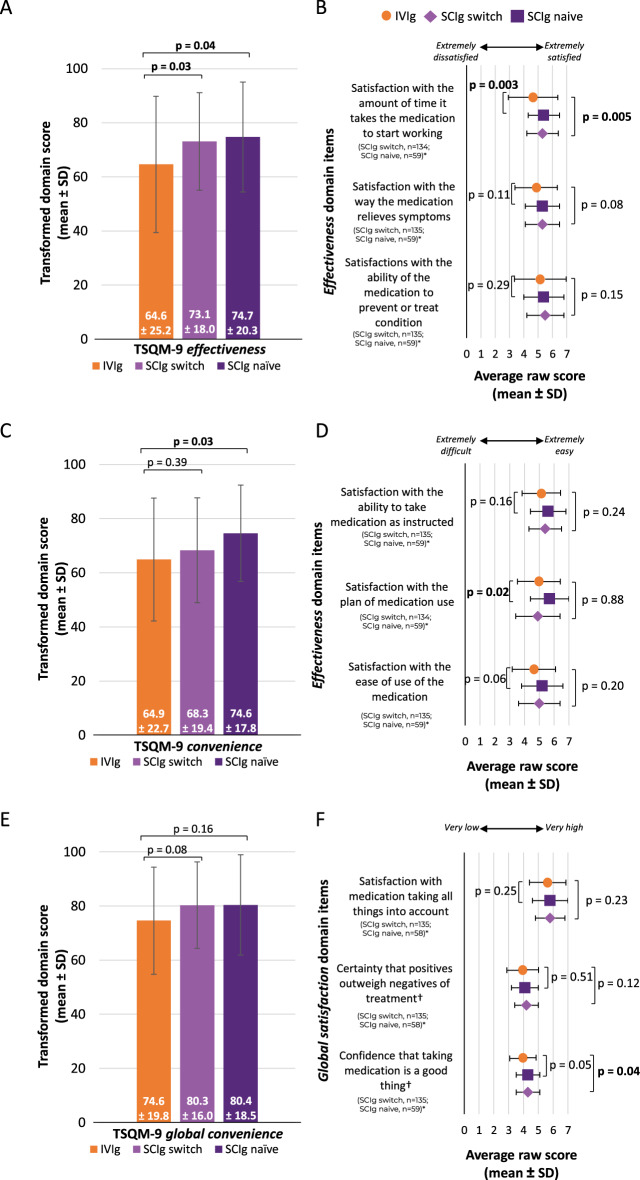
Perceived treatment satisfaction in the SCIg naïve and SCIg switch subgroups. **A** Transformed TSQM-9 *effectiveness* domain scores and **B** raw scores from the corresponding TSQM-9 domain items. **C** Transformed TSQM-9 *convenience* domain scores and **D** raw scores from the corresponding TSQM-9 domain items. **E** Transformed TSQM-9 *global satisfaction* domain scores and **F** raw scores from the corresponding TSQM-9 domain items. *n numbers vary due to missing respondent data for various survey questions. Transformed domain scores are on a 0–100 scale from worst to best and the raw scores are on a one to five or seven scale from extremely dissatisfied to extremely satisfied. Continuous variables were compared using an unpaired t-test and the significant p values are in bold. †Domain items rated on a scale from 1–5. *IVIg *intravenous immunoglobulin, *SCIg* subcutaneous immunoglobulin, *SD* standard deviation, *TSQM-9* treatment satisfaction questionnaire for medication 9

Compared with the IVIg cohort, both the SCIg naïve and SCIg switch subgroups had statistically significantly greater TSQM-9 *effectiveness* domain scores (p = 0.04 and p = 0.03, respectively; Fig. 5A). When examining item specific evidence in the TSQM-9 *effectiveness* domain, both the SCIg naïve and SCIg switch subgroups reported significantly greater satisfaction scores with *the amount of time it takes the medication to start working* compared with the IVIg cohort (p = 0.003 and p = 0.005, respectively; Fig. 5B).

##### TSQM-9 *convenience*

Compared with the IVIg cohort, the SCIg naïve subgroup had a statistically significantly greater TSQM-9 *convenience* domain score (p = 0.03, Fig. 5C). When examining item specific evidence in the TSQM-9 *convenience* domain, the SCIg naïve subgroup reported significantly greater satisfaction scores with* the planning of medication *use compared with the IVIg cohort (p = 0.02, Fig. 5D). Meanwhile, no significant differences in the TSQM-9 *effectiveness *domain score overall, or in any item specific evidence in this domain, were found between the SCIg switch subgroup and IVIg cohort (p > 0.05, Fig. 5C and D).

##### TSQM-9 *global satisfaction*

There was no significant difference between the SCIg naïve and IVIg cohorts in the score for the TSQM-9 *global satisfaction* domain (p = 0.16, Fig. 5E). Similarly, when examining item specific evidence in the TSQM-9 *global satisfaction* domain, no significant differences between the SCIg naïve subgroup and the IVIg cohort were found (p > 0.05, Fig. 5F). The SCIg switch subgroup scored numerically higher in the TSQM-9 *global satisfaction* domain compared with the IVIg cohort, but there was only slight evidence that the difference was significant (p = 0.08, Fig. 5E). When examining item specific evidence in the TSQM-9 *global satisfaction *domain, the SCIg switch subgroup reported significantly greater confidence scores *that taking medication is a good thing* compared with the IVIg cohort (p = 0.04, Fig. 5F).

#### Other patient-reported outcomes: differences between the two SCIg subgroups and the IVIg cohort

**Fig. 6:**
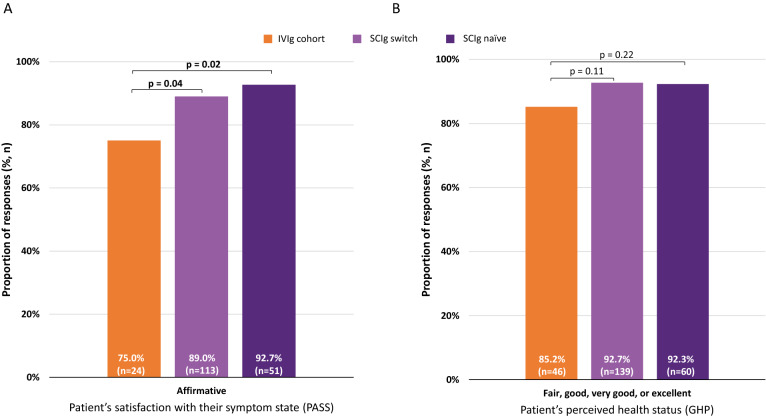
Respondent symptom state and perceived health status in the SCIg naïve and SCIg switch subgroups. **A** Proportion of respondents who responded ‘affirmative’ to whether they were at an acceptable symptom state (as measured using PASS) and **B** proportion of respondents who described their current health status as fair, good, very good, or excellent (as measured using GHP). The complementary response category for PASS was ‘negative’ and for GHP were ‘poor’ or ‘very poor’. Significant p-values are highlighted in bold. *GHP* general health perception, *IVIg* intravenous immunoglobulin, *PASS* patient acceptable symptom state, *SCIg* subcutaneous immunoglobulin

Overall, a higher proportion of both the SCIg naïve and SCIg switch subgroups perceived their current symptom state to be acceptable (via PASS) compared with the IVIg cohort (92.7% [n = 51] and 89.0% [n = 113], respectively, vs. 75.0% [n = 24]) (p = 0.02 and p = 0.04, respectively; Fig. 6A). In addition, measurement of overall well-being in terms of GHP revealed that lower proportions of both the SCIg naïve and SCIg switch subgroups reported very poor or poor health compared with the IVIg cohort (7.7% [n = 5] and 7.3% [n = 11], respectively vs. 14.8% [n = 8]; p = 0.22 and p = 0.11, respectively; Fig. 6B). There were no significant differences (all p > 0.05) between both the SCIg naïve and SCIg switch subgroups compared with the IVIg cohort for the mean (± SD) PROMIS GPH-2 T-scores (46.7 ± 8.1 [n = 58], and 47.4 ± 7.4 [n = 135], respectively vs. 46.3 ± 8.6 [n = 31], respectively) and GMH-2 T-scores (51.6 ± 6.8 [n = 58], and 50.9 ± 8.5 [n = 135], respectively vs. 51.3 ± 10.5 [n = 31], respectively).

## Discussion

In this analysis of a survey of Canadian, mostly Québec-based respondents with PID or SID, we evaluated the impact of IgRT infusion-related characteristics on patient-reported treatment satisfaction, symptom status, overall well-being, and physical and mental health. Specifically, we compared patient-reported treatment satisfaction between those receiving IVIg and those receiving SCIg, overall and stratified by whether the latter group was new to IgRT (SCIg naïve) or had switched from IVIg (SCIg switch). We also evaluated patient satisfaction in terms of its various domains (perceived *effectiveness*,* convenience*, and *global satisfaction*) as well as in terms of underlying items (questions).

In this study, respondents receiving SCIg reported significantly quicker infusion preparation time, actual infusion time, and post-infusion clean up time per infusion compared with IVIg users. Although SCIg infusions are typically more frequent than IVIg infusions, and total infusion time involvement over a monthly period may be the same, a recent study demonstrated that shorter infusion times per infusion were associated with substantially enhanced treatment satisfaction [30]. Given the apparent value placed by patients on shorter, flexible infusions, further infusion optimization could potentially help patients achieve more flexibility, reduce the time spent on their infusion schedule, and reduce the treatment burden of infusion regimens [31]. The median travel time for the collection of SCIg supplies was higher than the median travel time to receive infusions for IVIg respondents. However, the majority of SCIg respondents made fewer than one trip per month to collect their SCIg supplies, suggesting that respondents with longer journeys (for example, respondents who live far away from a pharmacy, or in rural locations) may prefer to pick up multiple months’ worth of supplies at once.

Overall, we found that SCIg respondents were associated with better treatment satisfaction (as measured by the TSQM-9) than IVIg respondents, in terms of perceived *effectiveness* in particular. This finding was supported by accompanying findings that a higher proportion of the SCIg cohort reported an acceptable symptom state and a lower proportion of the SCIg cohort reported they were in poor or very poor health, compared with the IVIg cohort.

It is notable that in this survey the prescriber was most likely to be responsible for choosing a respondent’s infusion method for both the IVIg and SCIg cohorts. Past studies have demonstrated that while patients value the ability to choose between treatment options, they also acknowledge their inability to make a completely informed choice due to their lack of clinical knowledge and, consistent with our findings, patient decision-making is largely influenced by the clinician or prescriber [40].

Despite prescribers playing a major role in IgRT modality choice, the finding that the SCIg cohort was associated with a better perceived *effectiveness* compared with the IVIg cohort may be, in part, attributed to the SCIg cohort (especially the SCIg naïve cohort) feeling more empowered. Self-infusing SCIg users play a more active role in their treatment compared with their IVIg counterparts, giving them the feeling of empowerment [41–44]. Indeed, patient preference assessments highlight the importance of continually offering patients information and a choice of IgRT infusion options [30–32].

Yet another additional or alternative explanation of better perceived *effectiveness* (as measured by the TSQM-9 *effectiveness* domain) may be that a more stable serum Ig profile can be achieved when using SCIg, due to greater frequency of infusions, compared with that achieved by IVIg [18–20], notwithstanding this being a misconceived reason for choosing IVIg among some participants in this survey. This interpretation appears supported by item specific evidence on the TSQM-9 *effectiveness* domain, for example, where SCIg patients reported significantly better scores on the ‘*amount of time it takes the medication to start working*’. Further, item specific evidence (from the TSQM-9 *global satisfaction* domain) found that SCIg patients reported higher scores on ‘*how confident are you that taking this medication is a good thing for you*’ compared with IVIg patients; again, these results support the notion of greater perceived empowerment among the former.

Overall, patients typically transitioned from IVIg to SCIg following prescriber recommendation, as previously noted, and reported it to be a generally positive experience, with many respondents reporting improved HRQoL, improved physical and mental health, improved productivity, and greater treatment satisfaction and compliance. Therefore, our findings provide further evidence demonstrating the positive impact reduced treatment complexity of SCIg can have on improving the overall quality of life and well-being of patients.

Among SCIg users, the most compelling evidence on treatment satisfaction came from patients who had no previous IVIg experience (SCIg naïve). This cohort of patients reported better perceived *effectiveness*, as well as better treatment *convenience*, compared with the IVIg cohort. The same was less true of those that had transitioned to SCIg from IVIg, and this may in part be due to them having encountered more barriers to transition to self-infusion (such as dose adjustments) or still being in the process of adjusting to the transition. Better treatment satisfaction among the SCIg naïve cohort was particularly remarkable considering their relatively short IgRT experience, since evidence suggests that treatment satisfaction is better among those with longer IgRT experience [30]. Accordingly, an alternative explanation for our finding could be that in the authors experience, many patients with immunodeficiencies start IgRT directly on SCIg in Canada, except those whose conditions are very unstable or have multifactorial obstacles (but are usually later switched to SCIg once stabilized).

Overall, the COVID-19 pandemic was found not to have impacted how respondents in Canada perceived their current IgRT infusion modality and did not cause many patients to switch IgRT infusion methods. Patients may have been resistant to changes in their treatment due to increased levels of health anxiety at the outset of the COVID-19 pandemic. Alternatively, patients may already have a relatively high level of satisfaction with their current IgRT infusion treatment method [19]. Nonetheless, the proportion of patients using SCIg in this study was four times more than a similar study of patients with PID using SCIg and IVIg in the US in 2017 [30], which may partly be attributed to the rise in popularity of home-based treatments in order to mitigate the risk of nosocomial infections during the COVID-19 pandemic, during which the survey was conducted. Cross-country differences in preference for and utilization of SCIg may also be a factor. In either case, it would be interesting to further evaluate if patients remained on SCIg or if patients returned to hospital-based IVIg as the risk of COVID-19 subsides.

Training patients for self-infusion with SCIg is documented to play an important role in patient reported infusion efficiency and satisfaction [30]. In this study, training experience varied within the overall patient population, with some patients finding the training more difficult or requiring more sessions. This finding could be explained by the fact that access to SCIg training in Canada may have been impacted during the COVID-19 pandemic [45, 46]. Improvements in SCIg training, such as trouble shooting and assessing for AEs, and regular monitoring following training, may result in higher rates of successful transitions to SCIg that are maintained long term [31].

Although not the focus of our survey, a number of studies have reported the economic benefits of SCIg over IVIg in patients with immunodeficiencies, for example one German cost minimization study found treatment with SCIg resulted in cost savings from the perspective of the German statutory health insurance system [47]. A UK review of IgRT evidence for patients with PID found home-based self-administration of SCIg to be cost-effective in comparison with hospital-administered IVIg [48]. Similarly, a French cost minimization simulation of patients with primary antibody deficiencies estimated savings of over €6,000 in switching from hospital-based IVIg and approximately €10,000 in switching from home-based IVIg to home-based SCIg [49]. A Canadian budget impact model of patients with PID projected savings of more than $5,000 to $8,000 over 3 years when patients are treated with SCIg rather than IVIg (depending on modality of the IVIg therapy) [50]. Finally, an Australian economic analysis of patients found that SCIg was associated with savings greater than $45,000 in PID and $6000 in SID over a 10-year period in comparison with IVIg [51, 52]. Taken together, home-based SCIg treatment has been found to provide cost savings in comparison with hospital and home-based IVIg treatment. The economic benefits, in addition to efficacy and safety benefits, can be used in parallel with evidence on PROs to provide a comprehensive picture of the IgRT administration options to choose from.

We acknowledge some limitations are inherent with patient-reported surveys and can result in potentially difficult interpretation. Responses could not be independently verified with patients’ physicians, so the results rely on accurate patient recall and understanding of the survey questions. Missing data points could also impart potential bias. However, the missing data observed in this survey is comparable to a previous study [30]. Further, in a cross-sectional survey such as this, no causation is implied between various patient-reported assessments. Indeed, choice of Ig modality may likely simultaneously have influenced various aspects of patient life. The survey was also limited to patients who were affiliated with Canadian organizations (i.e., CIPO and APIQ, who are predominantly established in Québec) and generalization of IgRT experiences to wider populations should be made with caution. Despite these potential limitations, our findings on variations in patient-reported treatment satisfaction across IgRT infusion methods will aid evidence-based decision making and ultimately improve patient outcomes.

## Conclusions

Patients with PID and SID receiving SCIg reported their actual infusions to be significantly quicker per infusion compared to those receiving IVIg. The SCIg cohort was associated with significantly higher scores for the TSQM-9 *effectiveness* domain compared with the IVIg cohort. Furthermore, a significantly higher proportion of SCIg patients reported their overall symptom state to be satisfactory compared with patients receiving IVIg. Certain SCIg subgroups, notably those initiating their IgRT treatment directly on SCIg, perceived their treatment to be more *effective* and *convenient* compared with respondents receiving IVIg. These findings improve our understanding of the overarching impact of different IgRT infusion methods on treatment satisfaction and well-being, and will hopefully allow for more evidence-based decision making to help guide best practice for IgRT and facilitate optimization of patient outcomes.

## Supplementary Information

**Additional file 1.** APIQ survey questions. The survey contained 101 questions on IgRT use and respondent perceptions. IgRT, immunoglobulin replacement therapy.

## Data Availability

The datasets analyzed during the current study are available from the corresponding author on reasonable request.

## Abbreviations

AEs: Adverse events
ANOVA: Analysis of variance
APIQ: Associatin des Patients Immunodéficients du Québec
CI: Confidence interval
CIPO: Canadian Immunodeficiencies Patient Organization
COVID-19: Coronavirus disease 2019
GHP: General health perception
GPH-2: Two-item Global Physical Health
GMH-2: Two-item Global Mental Health
HIV: Human immunodeficiency virus
HRQoL: Health related quality of life
HSCT: Hematopoietic stem cell transplantation
Ig: Immunoglobulin
IgG: Immunoglobulin G
IgRT: Immunoglobulin replacement therapy
IQR: Interquartile range
IVIg: Intravenous immunoglobulin
LQI: Life quality index
PASS: Patient acceptable symptom state
PIDs: Primary immunodeficiency diseases
PROMIS: Patient-reported outcomes measurement information system
PROs: Patient-reported outcomes
SCIg: Subcutaneous immunoglobulin
SD: Standard deviation
SF-36: Short-form 36 health status questionnaire
SIDs: Secondary immunodeficiency diseases
SOT: Solid organ transplant
TSQM: Treatment satisfaction questionnaire for medication
